# Correction to “Deep multimodal predictome for studying mental disorders”

**DOI:** 10.1002/hbm.26441

**Published:** 2023-07-29

Rahaman, M. A., Chen, J., Fu, Z., Lewis, N., Iraji, A., van Erp, T. G. M., & Calhoun, V. D. (2023). Deep multimodal predictome for studying mental disorders. *Human Brain Mapping*, *44*(2), 509–522. https://doi.org/10.1002/hbm.26077

In the originally published version of the article, the following funding information was missing from the Acknowledgments section:

**ACKNOWLEDGMENTS**

This study is funded by the National Institute of Mental Health (NIMH) grant numbers R01MH118695, R01MH123610.

